# The economic burden of bronchiectasis – known and unknown: a systematic review

**DOI:** 10.1186/s12890-019-0818-6

**Published:** 2019-02-28

**Authors:** Pieter C. Goeminne, Francisco Hernandez, Roland Diel, Anna Filonenko, Rowena Hughes, Fabian Juelich, George M. Solomon, Alex Upton, Kamonthip Wichmann, Weiwei Xu, James D. Chalmers

**Affiliations:** 1 Department of Respiratory Diseases, AZ Nikolaas, Sint-Niklaas, Belgium and Department of Respiratory Diseases, UZ Leuven, Leuven, Belgium; 2 Pharmerit, Rotterdam, The Netherlands; 3 Institute of Epidemiology, University Hospital Schleswig Holstein, Kiel, Germany; 4 Bayer AG, Berlin, Germany; 5 AccuScript Consultancy, Reading, UK; 6 Bayer Vital GmbH, Leverkusen, Germany; 7 University of Alabama at Birmingham, Birmingham, AL USA; 8 Bayer, Reading, UK; 9 Scottish Centre for Respiratory Research, University of Dundee, Dundee, UK

**Keywords:** Burden of illness, Bronchiectasis, Resource use, Costs, Hospitalization, Economic burden

## Abstract

**Background:**

The increasing prevalence and recognition of bronchiectasis in clinical practice necessitates a better understanding of the economic disease burden to improve the management and achieve better clinical and economic outcomes. This study aimed to assess the economic burden of bronchiectasis based on a review of published literature.

**Methods:**

A systematic literature review was conducted using MEDLINE, Embase, EconLit and Cochrane databases to identify publications (1 January 2001 to 31 December 2016) on the economic burden of bronchiectasis in adults.

**Results:**

A total of 26 publications were identified that reported resource use and costs associated with management of bronchiectasis. Two US studies reported annual incremental costs of bronchiectasis versus matched controls of US$5681 and US$2319 per patient. Twenty-four studies reported on hospitalization rates or duration of hospitalization for patients with bronchiectasis. Mean annual hospitalization rates per patient, reported in six studies, ranged from 0.3–1.3, while mean annual age-adjusted hospitalization rates, reported in four studies, ranged from 1.8–25.7 per 100,000 population. The average duration of hospitalization, reported in 12 studies, ranged from 2 to 17 days. Eight publications reported management costs of bronchiectasis. Total annual management costs of €3515 and €4672 per patient were reported in two Spanish studies. Two US studies reported total costs of approximately US$26,000 in patients without exacerbations, increasing to US$36,00–37,000 in patients with exacerbations. Similarly, a Spanish study reported higher total annual costs for patients with > 2 exacerbations per year (€7520) compared with those without exacerbations (€3892). *P. aeruginosa* infection increased management costs by US$31,551 to US$56,499, as reported in two US studies, with hospitalization being the main cost driver.

**Conclusions:**

The current literature suggests that the economic burden of bronchiectasis in society is significant. Hospitalization costs are the major driver behind these costs, especially in patients with frequent exacerbations. However, the true economic burden of bronchiectasis is likely to be underestimated because most studies were retrospective, used ICD-9-CM coding to identify patients, and often ignored outpatient burden and cost. We present a conceptual framework to facilitate a more comprehensive assessment of the true burden of bronchiectasis for individuals, healthcare systems and society.

**Electronic supplementary material:**

The online version of this article (10.1186/s12890-019-0818-6) contains supplementary material, which is available to authorized users.

## Background

Patients with non-cystic fibrosis bronchiectasis experience daily respiratory symptoms, such as chronic cough, sputum production, and exacerbations [1]. These symptoms cause significant morbidity, reducing physical performance, and severely affecting a patient’s health-related quality of life (HRQoL). Improving the quality of care for patients with bronchiectasis is paramount in limiting the impact of disease burden on daily functioning [2].

Management of bronchiectasis aims to control symptoms, reduce the incidence of exacerbations, and prevent disease progression. This is achieved through a multifaceted approach that includes airway clearance therapies, such as physiotherapy and/or exercise, antibiotic therapy, and anti-inflammatory treatment [1, 3–6]. In addition, hospitalization and antibiotic treatment may be required for the management of exacerbations. For these reasons, care is provided in both primary and secondary settings, and the provision of care may shift between these settings over the course of the disease. Indeed, the structure and intensity of healthcare resource use for the management of bronchiectasis may vary across different settings.

Although non-cystic fibrosis bronchiectasis has been a neglected disease, its apparent prevalence is increasing [7, 8], possibly reflecting improved diagnosis through greater use of high-resolution computed tomography (HRCT) of the chest [1, 3]. With the rising number of patients requiring appropriate management, a better understanding of the current economic disease burden of bronchiectasis is needed to ensure efficient allocation of healthcare resources. Therefore, the aim of this systematic literature review was to identify literature reporting resource use and costs associated with the management of bronchiectasis in adults, and to identify knowledge gaps. Based on the findings, we present a conceptual framework to facilitate a more comprehensive assessment of the true burden of bronchiectasis for individuals, healthcare systems, and society.

## Methods

A systematic literature review was conducted using MEDLINE, Embase, EconLit, and the Cochrane databases (Cochrane Database of Systematic Reviews, Database of Abstracts of Reviews of Effects, Cochrane Central Register of Controlled Trials, Health Technology Assessments Database, NHS Economic Evaluation Database, Cochrane Airways Group). The search aimed to identify publications reporting the economic and humanistic burden of bronchiectasis in adults. Searches included terms for bronchiectasis and terms relating to resource use, costs, cost-effectiveness and health-related quality of life (HRQoL).

The research covered the period from 1 January 2001 to 20 October 2015, and an update was carried out from 1 September 2015 to 31 December 2016. Searches were also performed to identify relevant abstracts presented at the following congresses (from October 2012 to January 2017): the European Respiratory Society, the Interscience Conference of Antimicrobial Agents and Chemotherapy, the European Congress of Clinical Microbiology and Infectious Diseases, the Infectious Disease Society of America (IDWeek), the British Thoracic Society, the International Society for Pharmacoeconomics and Outcomes Research, the American College of Chest Physicians (CHEST), and the First World Bronchiectasis Conference.

All electronic databases and congress searches underwent double-blind screening of the title and abstract by two researchers. Eligibility criteria included publications that reported costs, cost savings, and resource use in adults with bronchiectasis. Publications were excluded if they were in languages other English; reviews, editorials, notes and letters were also excluded. An example search string is included in Additional file 1. Selected articles underwent a full-text review to verify quality and eligibility. A random selection of excluded articles was reviewed by an independent researcher for quality-control purposes. Discrepancies between reviewers were discussed and amended.

All data were extracted into pre-defined data extraction grid by a single researcher. A second researcher independently checked all data. (See Additional file 2 for an example extraction grid).

## Results

**Fig. 1:**
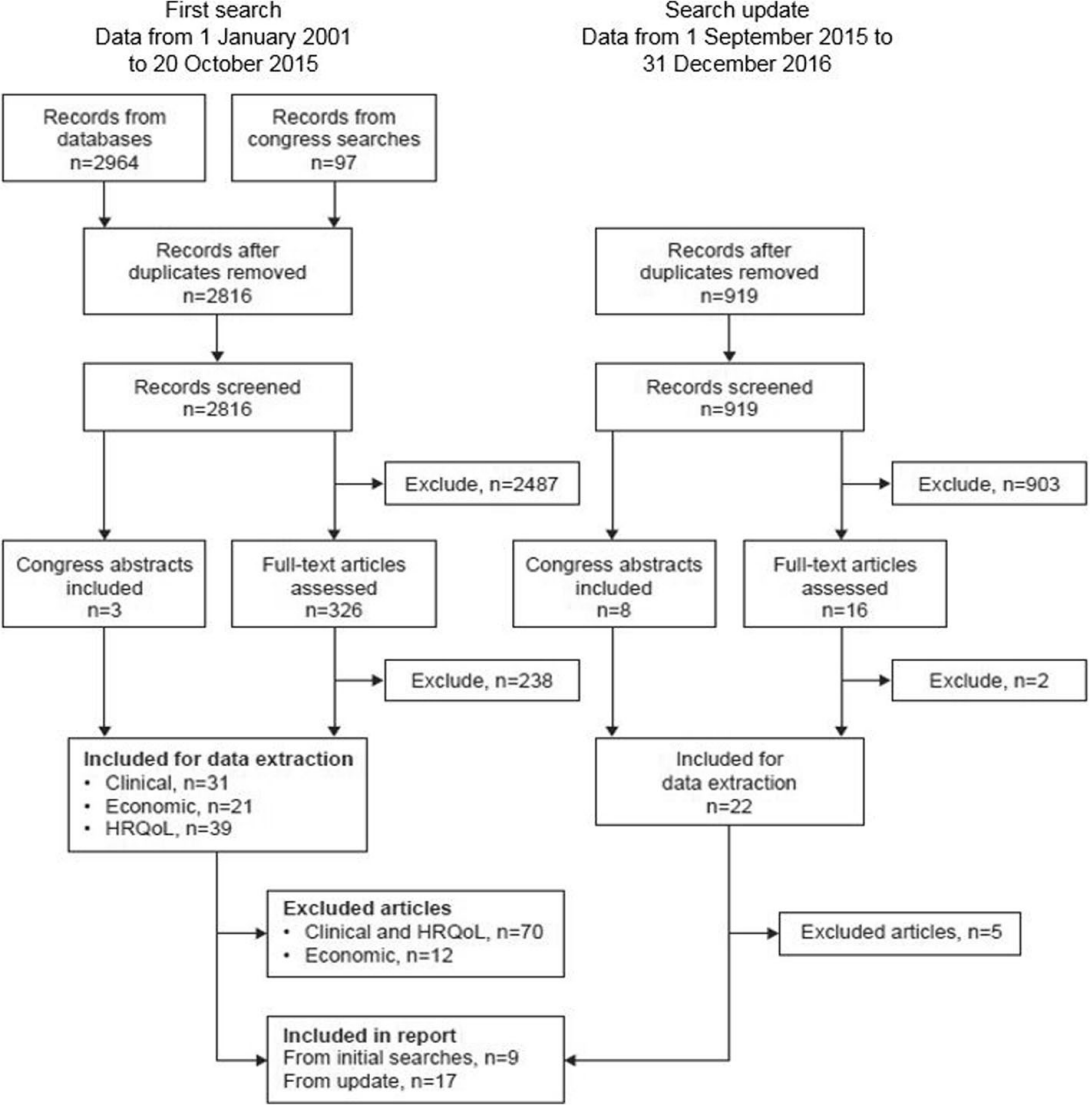
PRISMA flow diagram. Reasons for exclusion of articles from the initial search from inclusion in the report (*n* = 12): relevant data not reported or very limited (*n* = 4), patient population not specifically bronchiectasis (*n* = 5), and duplicate (full paper identified in the update search, *n* = 3). Reasons for exclusion of articles from the updated search: relevant data not reported or very limited (n = 5). The updated search identified economic and HRQoL studies but was only screened to identify economic articles

A total of 26 publications were identified reporting resource use and costs associated with the management of adults with bronchiectasis (see Fig. 1). One study identified as an abstract in the systematic review has subsequently been published as a full paper [9]. Fifteen studies included > 200 patients; of these, six were conducted in the USA [9–14], three in the UK [15–17], two in Spain [18, 19], and the others in New Zealand [20], Germany [21], Poland [22], and Singapore [23]. All studies involved adults and one included children [20] and a second included individuals of any age. [21] Most studies described in full papers reported on the presence of comorbidities, including cardiovascular disease, COPD, diabetes, respiratory disease, primary hypertension or reported a Charlson comorbidity index score, suggesting that many patients had comorbidities. Some comparative studies adjusted for comorbidities whereas others do not provide this level of detail in their description of the methodology used.

Resource use in the management of bronchiectasis was described in 24 studies. The following resource use items, indexed by setting, were reported:

- Inpatient care: hospital admission, including intensive care unit (ICU) and emergency room (ER) visits.
- Outpatient care: primary and secondary care visits, diagnostics (including an initial chest CT scan, blood tests, immunology tests, lung function tests) and/or monitoring by HRCT and microbiology, respiratory physiotherapy, and airway clearance techniques.
- Medication and other treatments: antibiotics, bronchodilators, corticosteroids, oxygen therapy.

Two US studies compared the resource use and costs for patients with bronchiectasis versus matched controls without bronchiectasis and provide an estimate of the incremental resource use and costs due to bronchiectasis. [12, 14]. Twenty-two further studies reported on hospitalization rates or length of stay for patients with bronchiectasis; [9, 11, 13–22, 24–33] some of these studies also reported on other aspects of resource use. Eight studies report the costs of managing bronchiectasis and provide evidence for cost drivers in this disease. [9, 10, 12, 14, 18, 19, 34, 35]

### Incremental resource use and cost due to bronchiectasis

Bronchiectasis is associated with increases in resource use and overall management costs compared with individuals without bronchiectasis, as demonstrated in two US studies. Weycker et al. performed a detailed evaluation of the costs of bronchiectasis based on an analysis of US claims during a 3-year period from January 1999 onwards [14]. Patients with bronchiectasis (*n* = 1424) were identified through ICD-9-CM codes and data collected included hospital admissions, outpatient visits, and prescription medications. Costs for patients with bronchiectasis were compared with matched controls within the claims database without bronchiectasis on the basis of age, sex, geographic region, and selected comorbid conditions. Compared with the controls, patients with bronchiectasis had on average longer hospitals stays (4.5 days vs 2.5 days), a greater number of admissions (0.6 vs 0.4), more outpatient encounters (20.1 vs 14.0), and more days of antibiotic, corticosteroid, and bronchodilator use (27.2, 12.2, and 4.5, respectively). Increased resource use resulted in an additional annual increment of US$5681 (95% CI US$4862–6593) compared with controls. Inpatient care accounted for 56% of the increase in costs; outpatient visits (16%) and outpatient prescriptions (18%) accounted for most of the remainder of the increased costs.

A more recent US MarketScan database analysis examined the increase in resource use and costs for patients with bronchiectasis during the first year after diagnosis [12]. Data were analyzed for a 5-year period from 2005 for patients diagnosed with bronchiectasis (*n* = 9146) and matched controls (without bronchiectasis) (*n* = 27,438). This study reported an annual incremental burden for patients with bronchiectasis vs controls of 2.0 more outpatient visits, 0.4 more ER visits, and 2.4 more pharmacy scripts (all *p* < 0.001). No increase in hospital admissions was observed. When only considering respiratory-related resource utilization, patients with bronchiectasis had 1.6 more outpatient visits, 0.3 more ER visits, and 0.4 more pharmacy scripts than controls (all *p* < 0.001). This resulted in an increase of US$2319 overall and US$1607 for respiratory-related costs for the first year after diagnosis.

### Hospitalization and other resource use

Twenty-two further studies reported hospitalization rates and/or the length of hospital stay in patients with bronchiectasis, likely reflecting management of severe exacerbations. Four studies used national databases in the USA (1993–2006), Germany (2005–2011), Spain (2004–2013), and New Zealand (2008–2013) to determine mean annual age-adjusted hospitalization rates for bronchiectasis as the primary diagnosis. Rates ranged from 1.8 to 25.7 per 100,000 population (Table 1) [13, 18, 20, 21]. In addition, two studies reported rates for bronchiectasis as any diagnosis; these rates were considerably higher (e.g., 9.4 vs 1.8 per 100,000 for Germany [21]) compared with those studies reporting rates of bronchiectasis as a primary diagnosis. Another six studies have reported mean annual hospitalization rates per patient in specific cohorts; values ranged from 0.3 to 1.3 (Table 1) [11, 19, 24–27].

**Table 1:** Hospitalization rates for bronchiectasis (mean age-adjusted and other rates)

Reference	Dates, N	Results
*Mean annual age-adjusted hospitalization rates*		
Bibby et al., 2015 [20] (New Zealand)	2008–2013 *N* = 5494	• 25.7 per 100,000 population (1° diagnosis)
Ringshausen et al., 2013 [21] (Germany)	2005–2011 *N* = 61,838	• 9.4 per 100,000 population (any diagnosis) • 1.8 per 100,000 (1° diagnosis)
Sanchez-Munoz et al., 2016 [18] (Spain)	2004–2013 *N* = 70,676	• 15.5 per 100,000 population (1° diagnosis)
Seitz et al., 2010 [13] (USA)	1996–2006 *N* = 258,947	• 16.5 per 100,000 population (any diagnosis) • 2.0 per 100,000 (1° diagnosis)
*Hospitalization rates*		
Chan et al., 2013 [24] (New Zealand)	2001–2008 *N* = 100	• Mean annual hospitalization rate per patient: 1.29
de Costa et al., 2015 [25] (Portugal)	2013–2014 N = 70	• Mean annual hospitalization rate per patient: 0.8
Hwang et al., 2013 [26] (South Korea)	NR *N* = 79	• Mean annual hospitalization rate per patient: 0.3 • Mean number of admissions among those who were admitted: 3.08
de la Rosa et al., 2016 [19] (Spain)	2013 *N* = 456	• Mean annual hospitalization rate per patient: 0.34 ± 0.9 • Mean annual hospitalization rate increased from 0.14 ± 0.5 per patient for patients with mild bronchiectasis to 1.05 ± 1.6 for those with severe bronchiectasis (*p* < 0.001)
Germino and Liao, 2016 [11] (USA)	2008–2012 *N* = 5847	• Mean inpatient visits in first year of follow-up: 0.32 per patient • A total of 22.2, 24.0, and 34.4% of patients with 1, 2, and 3 or more exacerbations had an inpatient visit
McDonnell et al., 2015 [27] (UK)	2007–2009 *N* = 155	• Mean annual hospitalization rate in 143 patients was 0.9 ± 1.6 per patient with self-reported total number of admissions equal to 131 with 90.8% admissions due to bronchiectasis
*Change in hospitalization rates with time*		
Navaratnam et al., 2014 [16]	2004–2011 NS	• 8611 and 15,885 hospitalizations for bronchiectasis in 2004 and 2011, respectively (1° diagnosis) • Overall annual increase in hospitalizations of 9% (RR 1.09; 95% CI, 1.08–1.10, *p* < 0.0001)
Navaratnam et al., 2015 [15]	2009–2013 *N* = 536	• 74 and 121 admissions to ICU in 2009 and 2013, respectively, equating to a crude annual increase of 8% (RR 1.08; 95% CI, 1.02–1.15, *p* = 0.01) (1° diagnosis)
Ringshausen et al., 2013 [21]	2005–2011 *N* = 61,838	• 8.9 and 10.6 hospitalizations per 100,000 in 2005 and 2011, respectively – an average increase of 2.9% (95% CI, 1.7–4.2, *p* < 0.00001) per year (any diagnosis) • 1.6 per 100,000 in 2005 to 1.8 per 100,000 in 2011 (from graph) (1° diagnosis)
Sanchez-Munoz et al., 2016 [18] (Spain)	2004–2013 N = 70,676	• 16.5 and 17.0 hospitalizations per 100,000 in 2004 and 2013, respectively (*p* < 0.001) (1° diagnosis)
Seitz et al., 2010 [13] (USA)	1996–2006 N = 258,947	• Annual percentage change was 2.4% for men and 3.0% for women, demonstrating an increase in the number of hospitalizations over the time period assessed (any diagnosis) • Increase of 1.7% for men and 2.6% for women (1° diagnosis)
Niewiadomska et al., 2016 [22]	2000–2011	• Crude hospitalization rate increased from 2.0 per 100,000 in 2000 to 8.1 per 100,000 in 2011 (1° diagnosis)

*1°* primary, *CI* confidence interval, *ICU* intensive care unit, *NR* not reported, *NS* not specified, *RR* relative risk

Six studies, conducted over the past 20 years, reported increases in hospitalizations over time (Table 1) [13, 15, 16, 18, 21, 22]. A UK study observed an annual increase in hospitalizations from 2004 (*n* = 8611) to 2011 (*n* = 15,885) of 9% (relative risk [RR] 1.09, 95% confidence interval [CI] 1.08–1.10, *p* < 0.0001) [16]. Another UK study reported a crude annual increase of 8% in admissions to the ICU for bronchiectasis as a primary diagnosis over a period of 5 years from 2009 [15]. The age-adjusted rate of bronchiectasis-associated hospitalizations also increased significantly in Germany over a similar period (2005–2011), from 8.9 to 10.6 per 100,000 population, corresponding to an average annual increase of 2.9% (95% CI 1.7–4.2, *p* < 0.00001) [21]. Similarly, a US study reported an annual increase of 1.7% for men and 2.6% for women for bronchiectasis as a primary diagnosis over a period of 10 years, from 1996 to 2006 [13].

Three studies demonstrated higher rates of hospitalization with increasing age [13, 20, 21], and four studies reported higher rates of hospitalization in women compared with men [13, 18, 20, 21]. For example, in Germany, Ringshausen et al. [21] observed the highest age-specific rate of hospitalization (for bronchiectasis as a primary or secondary diagnosis) of 39.4 per 100,000 population among men aged 75 to 84 years compared with 9.4 per 100,000 for all ages (overall study population). In a US study based on hospital discharge records (1993–2006), the annual rate of bronchiectasis-associated hospitalizations increased dramatically with age for both men and women; this was approximately 8-fold higher in patients aged 80–84 years compared with patients aged 55–59 years [13]. Hospitalizations per 100,000 population were also higher in women than men (20.6 per 100,000 vs 12.3 per 100,000).

Four studies reported a higher incidence of hospitalization in patients with *P. aeruginosa* than in patients without this infection [27, 31–33]. This included a large meta-analysis of 21 studies (performed in Europe, Asia, and Australia) that reported a 6.5-fold increase in hospital admission for patients with vs without *P. aeruginosa* (odds ratio [OR] 6.57, 95% CI 3.19–13.51, *p* < 0.0001) [31]. A further study [9] compared resource use for the 12 months before and following an index claim for *P. aeruginosa* in patients with bronchiectasis (*n* = 716). Statistically significant increases in the number of hospital admissions (3 pre- vs 7 post-*P. aeruginosa*), as well as ER visits (0.5 vs 1.0), office visits (16.3 vs 27.1), and pharmacy visits (23.2 vs 36.2), were observed following a diagnosis of *P. aeruginosa* infection (*p* < 0.0001 for all comparisons). Another study found that patients with extensive lung damage had a significantly greater risk of readmission compared with those with less extensive damage (*p* = 0.047) [28].

Another factor contributing to the need for hospitalization is the frequency of exacerbations. A US study (2008–2012) which analyzed MarketScan data on resource use in patients receiving treatment for exacerbations (*n* = 5847) found that the mean (± SD) annual number of hospital outpatient visits doubled from 10.8 ± 15.1 for patients with 1 exacerbation/year to 21.0 ± 22.3 in those with ≥3 exacerbations/year [11]. Similarly, mean (± SD) hospital length of stay increased from 6.9 ± 9.8 in patients with 1 exacerbation/year to 7.3 ± 6.9 and 9.3 ± 8.6 days, respectively, in patients with 2 and ≥ 3 exacerbations/year. Statistically significant increases in ER visits and physician office visits were also noted for patients with more frequent exacerbations (*p* < 0.0001).

The average length of hospital stay was reported in 12 publications [11, 13–18, 20, 25, 28–30] and ranged from 2 to 17 days. The annual number of exacerbations [11], the presence of comorbidities [28], and the extent of lung damage [28] were found to be associated with an increase in the length of hospitalization. Two other studies reported that *P. aeruginosa* infection was associated with a significantly longer duration of hospitalization [28, 33].

### Costs and cost drivers

Costs for the management of bronchiectasis have been reported in eight studies [9, 10, 12, 14, 18, 19, 34, 35] (see Table 2). A cost analysis of patients managed in six Spanish hospitals, described by de la Rosa et al. 2016 [19], reported a mean annual cost for bronchiectasis (including both hospital and primary care costs) of €4672 ± 6281 per patient (cost year 2013), increasing with disease severity (as measured by FACED score) from €2993 (score 0–2, i.e., low mortality risk) to €9999 (score 5–7, i.e., high mortality risk) [19]. The overall cost per patient is consistent with a second Spanish study, which determined the costs of bronchiectasis based on analysis of the Spanish National Hospital Database [18]. The mean annual cost for 2013 was estimated at €3515 for patients with bronchiectasis as a primary diagnosis (*n* = 70,676) and €4559 for patients with a secondary diagnosis (*n* = 211,531). Four other studies report the annual costs of bronchiectasis in the USA, which ranged from US$13,244 (cost year 2001) [14] to US$26,284 (in patients without exacerbation, 2008–2011) [34, 35], US$37,030 (in patients with exacerbation, 2008–2011) [34], and US$67,764 (in patients with *P. aeruginosa,* 2007–2013). [9]

**Table 2:** Total annual cost per patient for bronchiectasis

Reference	Time frame (cost year)	Annual cost per patient	
De la Rosa et al., 2016 [19] (Spain)	2013 (2013)	Overall €4672 ± 6281 FACED 0–2: €2993 FACED 3–4: €4732 FACED 5–7: €9999	
Sanchez-Munoz et al., 2016 [18] (Spain)	2004–2013	Bronchiectasis 1° diagnosis 2004: €3961 2013: €3515 Time trend: *p* < 0.001	Bronchiectasis 2° diagnosis 2004: €4327 2013: €4559 Time trend: *p* < 0.001
Weycker et al., 2005 [14] (USA)	1999–2002 (2001)	Bronchiectasis: US$13,244 Control: US$7563 Incremental cost for bronchiectasis: US$5681 (95% CI US$4862–6593)	
Joish et al., 2013 [12] (USA)^a^	January 2005 to December 2009	Overall incremental cost of bronchiectasis: US$2319 (95% CI US$1872–2765)	Respiratory-related incremental cost of bronchiectasis: US$1607 (95% CI US$1406–1809)
Joish et al., 2013 [34] (USA)^a^	July 2008 to July 2011	Annual cost of bronchiectasis with exacerbation, US$37,030 Annual cost of bronchiectasis without exacerbation, US$26,284 Incremental cost, adjusting for baseline expenditure, US$8120 Incremental hospital costs: US$6147	
Joish et al., 2013 [35] (USA)^a^	July 2009 to July 2010	Annual cost of bronchiectasis with exacerbation, US$35,718 Annual cost of bronchiectasis without exacerbation, US$26,868 Incremental cost, adjusting for baseline expenditure: US$7643 Incremental hospital costs: US$5772	
Blanchette et al., 2017 [9] (USA)^b^	2007–2013	Total healthcare cost per patient: For year before *P. aeruginosa* diagnosis: US$36,213 For year after *P. aeruginosa* diagnosis: US$67,764 Incremental cost: US$31,551	
Blanchette et al., 2016 [10] (USA)^b^	2007–2013	Adjusted total cost Incremental increase in patients with *P. aeruginosa*: US$56,499 Incremental hospital costs: US$41,972	

*CI* confidence interval

^a^Based on data from the MarketScan claims database

^b^Based on data from the PharMetrics Plus administrative claims database

**Fig. 2:**
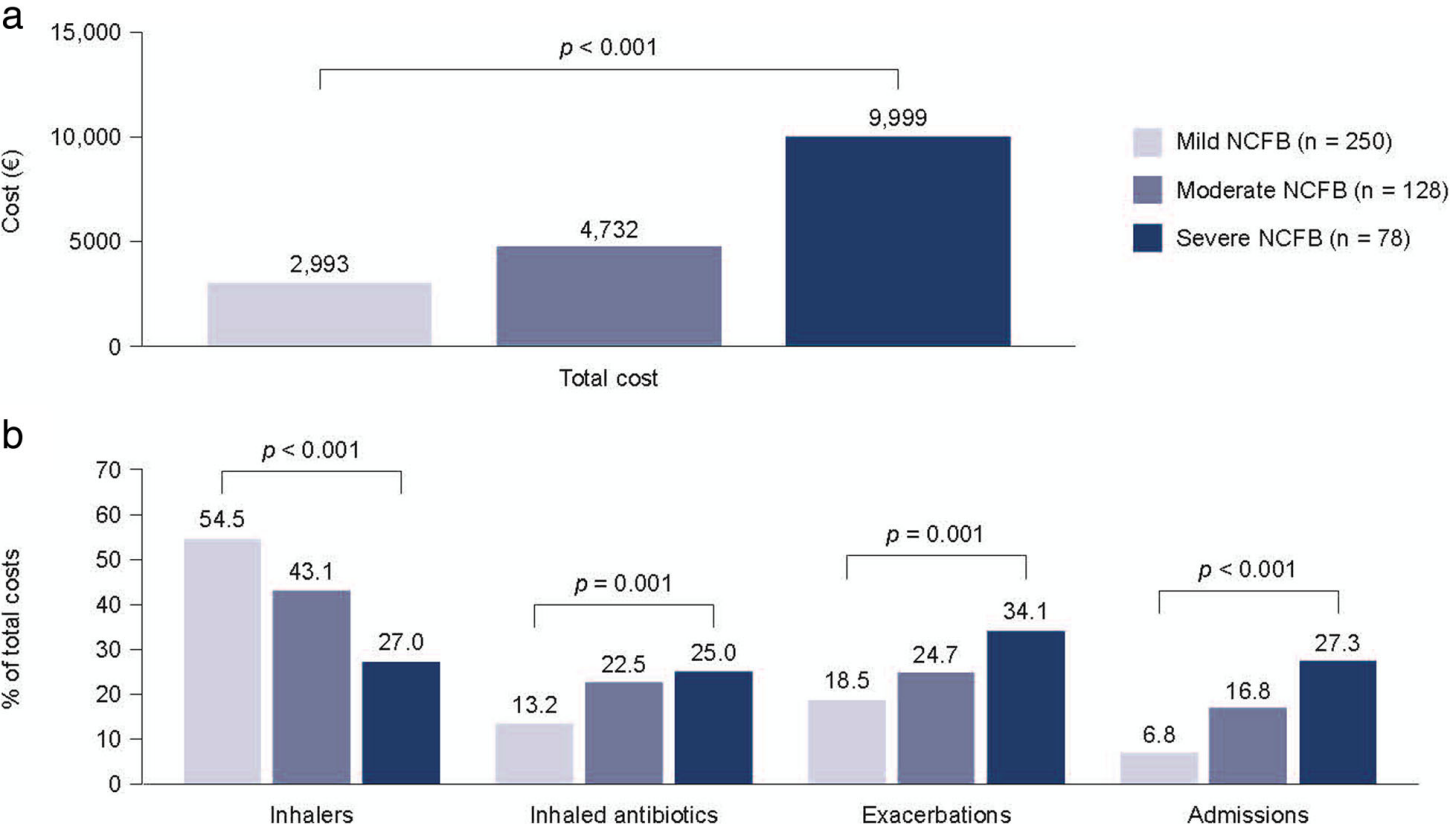
Distribution of costs in patients with bronchiectasis according to disease severity. Analysis of data from six hospitals (*n* = 456). Severity was assessed according to the FACED score: mild, 0–2 (i.e. low mortality risk); moderate, 3–4; and severe, 5–7 (i.e. high mortality risk) Inhalers included long-acting beta-agonists, inhaled corticoids and short- or long-acting anticholinergics. Adapted from de la Rosa et al., 2016 [19]

De la Rosa et al. [19] also reported on the elements contributing to the management costs for bronchiectasis and factors associated with increased costs. Overall, bronchodilators, corticosteroids, and short- or long-acting anticholinergics accounted for the largest proportion of total costs (46%). However, as these agents are not recommended for bronchiectasis, these costs may relate to the management of comorbidities such as chronic obstructive pulmonary disease (COPD), or inappropriate use in patients with bronchiectasis. Exacerbations (23%), inhaled antibiotics (18%), and admissions (13%) accounted for the remainder of the total costs. In patients with a high FACED score (i.e., greater risk of mortality), exacerbations accounted for the greatest proportion of the total costs (34%) whereas in patients with a low FACED score, exacerbations accounted for only 18.5% of the total costs (Fig. 2). Similar differences across the three severity cohorts were observed for the proportion of costs relating to inhaled antibiotics and admissions. In contrast, inhalers (long-acting beta-agonists, inhaled corticosteroids, and short- or long-acting anticholinergics) accounted for 54.5% of total costs for patients with a low FACED score compared with 27.0% of total costs for patients with a high FACED score (Fig. 2). Hence, the costs of managing exacerbations increased dramatically in patients with severe disease (from €769, FACED score 0–2 to €4305, FACED score 5–7). Total annual costs were higher in patients experiencing > 2 exacerbations per year compared to ≤2 exacerbations (€7520 vs €3892, respectively), and in patients with versus without COPD (€7448 vs €4168, respectively). However, patients experiencing > 2 exacerbations a year, or having severe disease, accounted for only approximately 20% of the total population. Therefore, this study found that a disproportionately large proportion of the cost of bronchiectasis relates to management of patients with severe disease.

Other studies have also reported that managing exacerbations is a significant cost driver. Two US studies reported an incremental increase in annual overall costs for patients with exacerbations of US$8120 and US$7643 [34, 35]. Two further US studies report an increase in costs for patients with *P. aeruginosa* infection of US$41,972 and US$31,551. [9, 10]

## Discussion

The published literature suggests that hospitalization costs constitute a major economic burden associated with bronchiectasis, especially in patients who experience frequent exacerbations. This is reflected in the results of several studies analyzing data from US claims databases for patients with bronchiectasis [9, 10, 14]. Consistent with this, an assessment of hospitalization rates and lengths of hospitalization, based on US claims, has shown an increase in resource use with frequency of exacerbations [11]. In addition, two economic assessments have reported an increase in annual overall costs of approximately US$8000 for patients experiencing exacerbations compared with those without exacerbations [34, 35]. While data for Europe and the US cannot be compared directly given the difference in currency and the cost years of the different studies, comparison of the annual costs reported for Spain [18, 19] (€4000 in 2013, corresponding to approximately US$5000 at current exchange rates) and the USA [34, 35] (US$26,000 to 35,000 for 2007–2013) suggest that costs are considerably higher in the USA. This may reflect higher rates of hospitalization in the USA but also differences in design between the studies. However, clinical experience clearly indicates that hospitalization for the treatment of exacerbations merely represents part of the management of patients with bronchiectasis. Hence, the true economic burden of bronchiectasis is not well reflected when only hospitalization or associated resource use is taken into consideration. While hospitalization plays an important role in the management of exacerbations, most patients require ongoing treatment for symptoms throughout the course of their disease, much of which is given in primary care. Furthermore, the introduction of new treatments that improve the management of bronchiectasis may reduce not only the need for hospitalization but also care in outpatient and primary care settings.

In addition to demonstrating the contribution of hospitalization to the overall costs of managing bronchiectasis, the identified studies showed that costs increase with the severity of disease and are higher in patients with *P. aeruginosa*. This is consistent with the observation that such patients are sicker and, independently of other factors, have increased hospitalizations [36] – the major driver of healthcare costs – and worse quality of life. Therefore, the evidence supports a conclusion that *P. aeruginosa* itself is a driver of increased healthcare costs. The increased costs associated with disease severity and *P. aeruginosa* infection emphasize the importance to the healthcare system of improving management of these patients. Current management for bronchiectasis patients is suboptimal compared with guideline recommendations [6] as illustrated in one study [37]. From a healthcare system perspective, initiatives to improve quality and cost-effectiveness of care require first an understanding of the burden of disease and associated costs.

The predominance of hospitalization and related resource use in the economic burden of bronchiectasis according to the published literature may reflect the types of studies and the information available from retrospective analyses. This may incompletely reflect many aspects of the economic burden of bronchiectasis, such as costs and resource use in primary care. Even the most comprehensive study identified in this review [19] may have overemphasized the role of hospital-based treatments because the cohort was based on patients attending specialist hospital clinics and not regular outpatient settings.

These retrospective studies rely on accurate recording of a bronchiectasis diagnosis in patient records or claims, e.g., using the ICD-9-CM codes. Similarities between bronchiectasis-related symptoms and other better recognized respiratory diseases, such as COPD and asthma, and greater availability of treatments for these respiratory conditions, can lead clinicians to miscode bronchiectasis. Therefore, using patient records or claims are likely to underestimate the real prevalence of bronchiectasis and the associated resource use.

A more accurate evaluation of the burden of bronchiectasis should take into account both the substantial impact of bronchiectasis on patients and the costs and resource use involved in treating patients across the whole spectrum of the disease. Indirect and intangible costs associated with bronchiectasis are likely to include the impact of ongoing symptoms and exacerbations on patient HRQoL and well-being. These costs should be considered both in the early stages of disease and through the changes to advanced disease. While the impact of bronchiectasis on HRQoL and well-being has been documented, [38–42] other intangible costs have not been studied. For example, the impact of ongoing symptoms on work productivity should be further explored. Clinical experience indicates that some patients have to reduce their working hours, or take early retirement, while others may be less productive because of the effects of symptoms. However, absenteeism, a reduction in working hours, or early retirement are difficult to estimate in patients, their families, and caregivers. To our knowledge, these aspects have not been analyzed, or reported. Furthermore, physiotherapy and regular airway clearance may require substantial time investment from patients on a daily basis. Other costs that may arise from severe disease include adaptations to the house, or the need to move into a nursing home, but evidence for such indirect and intangible costs has not been systematically reported.

In addition to the indirect and intangible costs of bronchiectasis, clinical experience clearly indicates that bronchiectasis is associated with extensive direct medical costs that go far beyond those documented in the publications identified in this review. For instance, from our sample of studies, we observed that costs for diagnostic tests, or the extensive range of interventions and monitoring, often initiated in primary care (e.g., physiotherapy, pulmonary rehabilitation, and sputum microbiology), have not been reported. Costs related to secondary care have also been omitted, such as providing home intravenous and nebulized antibiotic therapies, nutritional interventions, and radiologic assessments [1, 3–5]. Social care is also an important part of patient management and should also be studied. Advanced disease may necessitate home oxygen therapy, regular hospital admissions, and other costly interventions, such as lung transplantation [1, 3–5]. Self-payment costs for medical treatments and procedures could be substantial and have been reported by patients from several countries. However, these costs are not well documented or reported.

## Conclusions

The results of this systematic review indicate that the costs of management of bronchiectasis are substantial and are likely to be underestimated in the current published literature. Most studies are specific to the US healthcare landscape and have focused on the management of exacerbations. Only two studies reported the economic burden of bronchiectasis in Europe, namely in Spain. Assessing the burden of bronchiectasis is complex given the wide range of interventions used across different settings and across the spectrum of disease severity, together with the impact of comorbidities, such as anxiety, depression, cardiovascular disease and other respiratory diseases. More accurate assessments are needed to ensure appropriate allocation of resources to enable patients to receive optimal care and to enable accurate assessment of the cost-effectiveness of new interventions. Such assessments need to take into account the full spectrum of interventions and their roles across the clinical pathway for the management of bronchiectasis, and the impact of symptoms and treatments on the HRQoL and productivity of patients and carers.

**Fig. 3:**
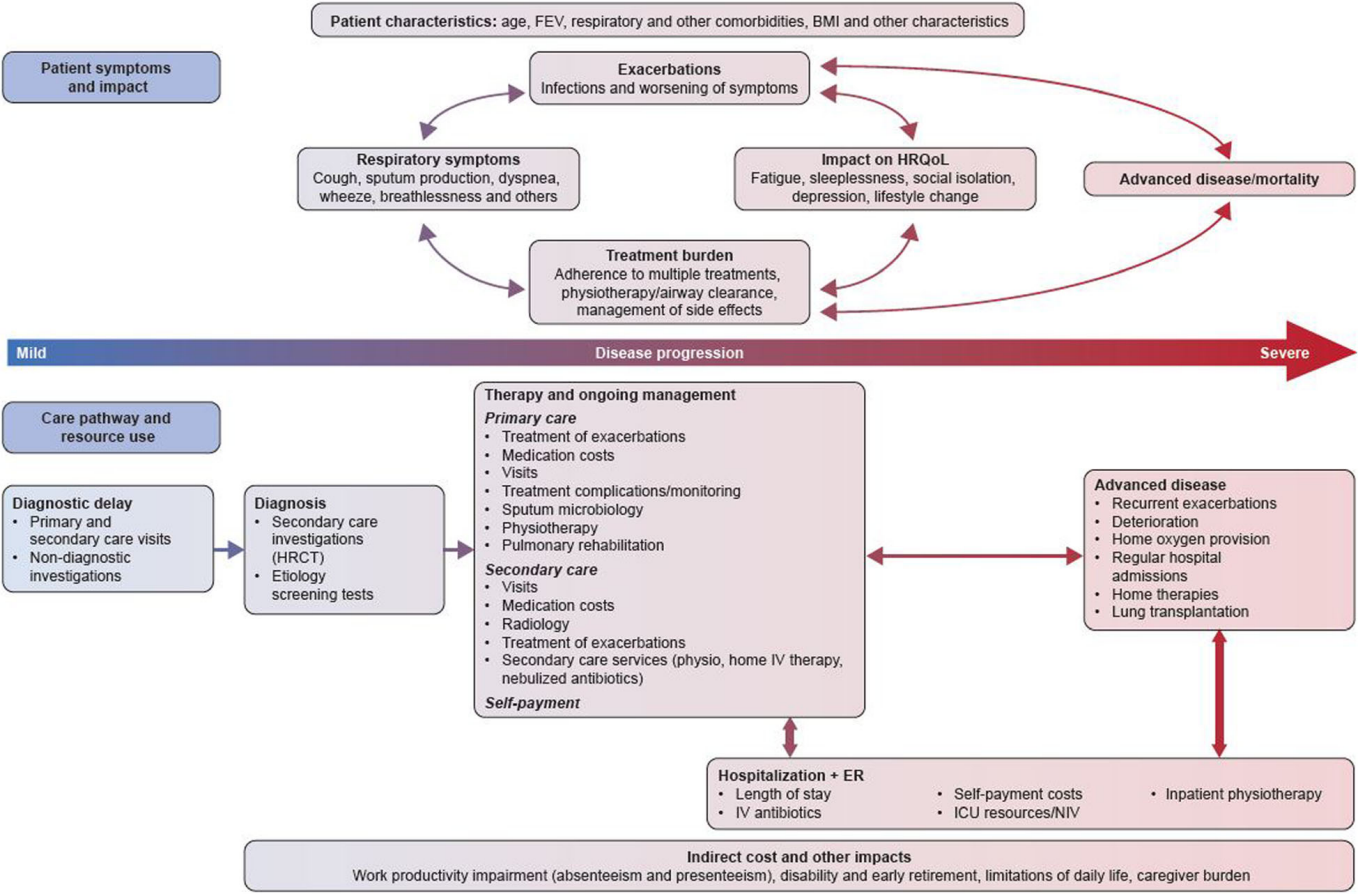
Conceptual framework for assessment of the economic burden of bronchiectasis. *BMI* body mass index, *ER* emergency room, *FEV* forced expiratory volume, *HRCT* high-resolution computed tomography, *HRQoL* health-related quality of life, *ICU* intensive care unit, *IV* intravenous, *NIV* non-invasive ventilation

For this reason, as outlined in Fig. 3, we propose a conceptual framework that could be used for future research, including prospective and retrospective observational studies, and economic models to facilitate the comprehensive estimation of the burden and impact of bronchiectasis in different healthcare settings. While the treatment pathway is largely comparable in most developed countries, differences will remain regarding the settings and costs of interventions. Evaluations in different countries may therefore be necessary. Considering patients across the whole disease spectrum, the impact of comorbidities, and the impact of bronchiectasis on other conditions will also be important to comprehensively map the economic burden of bronchiectasis.

## Additional files

Additional file 1: Example search string. Search terms ProQuest – Health-economic Evidence (searched on 20/10/2015) (DOCX 18 kb)

Additional file 2: Sample of extraction grid. Grids for capturing the data extracted (PDF 50 kb)

## Abbreviations

CI: Confidence interval
COPD: Chronic obstructive pulmonary disease
ER: Emergency room
HRCT: High-resolution computed tomography
HRQoL: Health-related quality of life
ICU: Intensive care unit
OR: Odds ratio
RR: Relative risk
SD: Standard deviation
SF-36: 36-Item Short Form Survey
SGRQ: St George’s Respiratory Questionnaire
