# Supplementary material for: The economic burden of bronchiectasis – known and unknown: a systematic review
Source: BMC Pulm Med. 2019 Feb 28;19:54. doi: 10.1186/s12890-019-0818-6 (PMC6393984; doi:10.1186/s12890-019-0818-6)
Supplement: Supplementary file 2 — Sample of extraction grid. Grids for capturing the data extracted (PDF 50 kb) [file 12890_2019_818_MOESM2_ESM.pdf]

		Publication												Patient population			
# ID	Intervention	Authors	Year	Title	Journal	Volume	Issue	Pages	Abstract	Country	Treatment setting	Study design	Main conclusions	Inclusion/exclusion criteria			
														Inclusion criteria	Exclusion criteria	Age (for inclusion)	FEV1 (for inclusion)

						Costs								Resource utilization (mean, SD, per p
	Patient characteristics													
Exacerbations (yes/no)	N	Age (mean, SD)	Gender (male: %)	Comorbidity	Disease state	Currency, year	Costs per episode of hospitalization	Costs per treatment cycle	Costs per month	Costs per year	Miscellaneous costs (drug costs, hospitalization costs, GP/specialists vist costs, etc.) *per year - specify cost category*	Miscellaneous costs (drug costs, hospitalization costs, GP/specialists vist costs, etc.) *per month - specify cost category*	Other costs	Frequency of GP visits *(specify per month or per year)*

atient)								Comment
Frequency of specialists visits *(specify per month or per year)*	Frequency of hospitalization *(specify per month or per year)*	Length of hospitalization (mean, SD)	Frequency of ER visits *(specify per month or per year)*	Frequency of radiological examinations, including CT *(specify per month or per year)*	Frequency of laboratory testing *(specify per month or per year)*	Drug dosage *(specify per day)*	Other resource utilization	
